# Introduction to the Special Issue of *Plants* on “Advances in Plant Reproductive Ecology and Conservation Biology”

**DOI:** 10.3390/plants13050605

**Published:** 2024-02-23

**Authors:** Brenda Molano-Flores, James I. Cohen

**Affiliations:** 1 Illinois Natural History Survey, Prairie Research Institute, University of Illinois Urbana-Champaign, 1816 South Oak Street, Champaign, IL 61820, USA; 2 Department of Botany and Plant Ecology, Weber State University, 1415 Edvalson St., Dept. 2504, Ogden, UT 84408, USA

Plant reproductive ecology explores aspects of the biology and ecology of plants ranging from breeding systems, plant–pollinator interactions, seed germination, floral traits, and much more. Plant conservation biology is an interdisciplinary field encompassing plant reproductive biology, population genetics, systematics, modeling, management, and policy, among others. This Special Issue on “Advances in Plant Reproductive Ecology and Conservation Biology” focuses on three main areas of research: population genetics, breeding systems, and ecology of common and rare plants. Each paper provides insights into new discoveries associated with these themes based on a wide variety of methods, with conservation biology being a thread that ties all of these studies together. Lastly, this Special Issue brings research voices from five of the seven continents, and from the USA which, in 2023, celebrated 50 years of the Endangered Species Act of 1973 [1]. We have received papers from California, Tennessee, and Florida, hotspots of biodiversity in North America. Below we provide a synopsis of each paper.

Eight studies in the Special Issue use population genetic methods and demonstrate that these approaches remain useful for exploring conservation biology. Each tried to gain a better understanding of rare species using a distinct approach. Tsaballa et al. [2] and Mansour et al. [3] investigated economically important species that have been impacted by human development. Tsaballa et al. [2] employed molecular barcoding to uncover the identity of wild species of orchids in Salep, a powder used in beverages and food for medicinal purposes that is culturally important in the eastern Mediterranean, and this has resulted in the overharvesting of orchids. The authors recognized that Salep, in Greece, is composed of species from four genera, with the greatest percentage from *Dactylorhiza* Neck. ex Nevski (Orchidaceae), an unexpected result given that species of *Orchis* L. (Orchidaceae) produce superior Salep. Barcoding is useful in this case as it provides evidence that overharvesting has resulted in a shift in the species used for Salep production. Mansour et al. [3] also examined a species, *Commiphora gileadensis* (L.) C.Chr. (Burseraceae) used by humans for perfume and medicine. Using microsatellites, the researchers found that even though the number of populations has declined, genetic diversity can still be recognized, and this diversity is not geographically structured, possibly due to human activity in Saudi Arabia.

Morris et al. [4], using microsatellite loci, and Cantley et al. [5], Hanko et al. [6], Cohen and Turgman-Cohen [7], and Guilliams and Hasenstab-Lehman [8], employing single nucleotide polymorphisms (SNPs) generated via reduced representation sequencing methods [9,10], elucidated patterns of genetic diversity for rare plant species. Chamorro-Premuzic et al. [11] studied *Dalea foliosa* (Gray) Barneby (Fabaceae), a species restricted to populations in Alabama, Illinois, and Tennessee, USA. These authors found that the majority of the genetic diversity was present in the center of geographic diversity, Tennessee, and nearby populations tended to be genetically similar in this area; however, populations in Alabama and Illinois were quite homogenous, suggesting that genetic diversity decreases toward the edges of the geographic range of the species. These authors also used ecological niche modeling to suggest that at present the niche for the species may be wider than observed currently, but that by 2070, this will have shrunk considerably, which can result in an issue for long-term persistence for the species. Cantley et al. [5] examined species of *Solanum* L. (Solanaceae) in the Australian Monsoon Tropics. These authors compared dioecious and cosexual species and found that while dioecious species had greater genetic diversity compared to cosexual ones, all populations of the five studied species had high rates of inbreeding. Hanko et al. [6] studied a rare species, *Scutellaria floridana* Chapm. (Lamiaceae), that frequently reproduces sexually and asexually. The researchers identified low overall genetic diversity across the species, which was, in part, the result of clonal reproduction; however, some populations harbored higher levels of genetic diversity providing the species with population structure. Cohen and Turgman-Cohen [7] investigated the Great Lakes endemic, *Iris lacustris* Nutt. (Iridaceae), a species that previously had been demonstrated to have limited genetic diversity across its geographic range [12]. Using SNPs, these authors not only recognized genetic diversity and population structure across the northern Great Lakes, but also hypothesized a pattern of western to eastern migration for the species. Guilliams and Hasenstab-Lehman [8] examined a rare species of *Eriodictyon* Benth. (Namaceae), *E. capitatum* Eastw., restricted to western Santa Barbara County in southern California. Most of the studied populations were quite genetically homogenous, which may be due to the clonal growth of the species. A phylogenomic analysis of *Eriodictyon*, also based on SNPs, recovered *E. capitatum* as monophyletic and as sister to *E. altissimum* P.V. Wells. Both species bear narrow leaves, an uncommon feature in the genus.

Among these eight studies focusing on population genetics, two common threads are woven. First, the breeding system and human influence are important in the current population structure of the species. This ranges from the orchid species used in Salep to the role of climate change in the shifts in weather in Australia to the modifications in the fire regime in south Florida [2,5,6]. Second, the hypothesized patterns of genetic diversity were not often recovered. Greater than anticipated genetic variation was found in *S. floridana*, *I. lacustris*, and *E. capitatum*, but only minimal differences were identified between dioecious and cosexual species of *Solanum*, *C. gileadensis*, *D. foliosa*, and for species used in Salep [2,3,5,6,7]. Collectively, these studies demonstrate the important role that field and laboratory studies play in ensuring a comprehensive understanding of population biology.

Five studies in the Special Issue examined aspects of plant breeding systems to illuminate conservation biology of diverse taxa. Pimienta and Koptur [13] examined the sphingophilous *Guettarda scabra* (L.) Vent. (Rubiaceae) and found that although the species has nocturnal flowers, the flowers remain open into the early morning, which allows for the plant to play host to a larger arthropod community. Ramalde et al. [14] explored dioecy in *Diospyros sericea* A.DC. (Ebenaceae), and these authors not only recognized vestigial sexual organs in the unisexual flowers but also identified some plants as being sexually leaky, with plants that develop staminate flowers also producing some fruits. In multiple species of *Iris*, Lozada-Gobilard et al. [15] investigated the role of visual floral cues as honest signals. The researchers found evidence of honest signaling, across multiple species of *Iris*, in a garden setting, but the effect was population-specific in natural environments, with abiotic factors possibly playing a role. In the rare *Macbridea alba* Chapm. (Lamiaceae), Johnson et al. [16] surveyed seven populations of the species. While seed production varied across the populations, with two having the majority of the seed output, overall seed production was low. The authors attributed this to multiple factors, including a small number of floral visitors and seed herbivory, a factor that had not been previously identified. Across three species of *Sorbus* L. (Rosaceae) endemic to eastern Slovakia, Kolarčik et al. [17] recognized tetraploid and triploid species and found that while seed production was uncommon, rare fertilization events were necessary and sufficient to retain the long-term viability of the small populations of these endemic species.

These studies on breeding systems collectively point to the need to understand the myriad manners in which plants reproduce in order to develop successful conservation biology projects. Additionally, and possibly more importantly, these studies demonstrate that it is crucial to take a closer look at supposedly understood biological phenomena. For example, Ramaldes et al. [14] recognized fruit development on staminate plants of the dioecious *D. sericea*; Pimienta and Koptur [13] identified new floral visitors for *G. scabra* given that the plants were open during dawn, not just at night; Johnson et al. [16] recognized seed herbivory as a potential limiting factor in the success of *M. alba*; and Kolarčik et al. [17] found the critical role rare reproductive events play in maintaining species. These studies on plant breeding systems should serve as a reminder to botanists and conservation biologists of the important role of careful field observations.

Lastly, three studies in the Special Issue focused on several aspects of the ecology of three rare species. Wadl et al. [18] provide a comprehensive review of the research accomplished with the endangered *Pityopsis ruthii* (Small) Small (Asteraceae), endemic to a small geographic area in Tennessee, USA. A collaborative research team has worked for almost three decades to better understand the biology and ecology of this species and factors that could increase its vulnerability to extinction. This paper also highlights the value of partnerships between researchers and state and federal agencies as an integral component for the conservation of the species. Work by Lange et al. [19] focuses on another USA endangered taxon *Sideroxylon reclinatum* Michx. subsp. *austrofloridense* (Whetstone) Kartesz and Gandhi (Sapotaceae). In this paper, the authors used microscopy work to identify morphological differences between the rare cryptic *Sideroxylon reclinatum* subsp. *austrofloridense* and *Sideroxylon reclinatum* subsp. *reclinatum*, the more common subspecies. In addition, the authors developed habitat suitability models (HSMs) for the species. Field verification of the HSMs in search of new populations of that rare cryptic species and new morphological characters to identify the subspecies will assist with identification and protection of critical habitat designation for this rare taxon, even when hybridization is a concern between the two subspecies. Lastly, Heineman et al. [20] focus on the annual USA federally threatened *Acanthomintha ilicifolia* (A. Gray) A. Gray (Lamiaceae). The researchers conducted a common garden study to better understand climate change stressors (e.g., water availability) and adaptability across the range of the species. Focusing on aboveground growth (biomass, height, and width) and reproductive output (flower number, seed number, and seed viability), the study highlights the role of local adaptation in species’ responses to climate change. These studies are a reminder of the value of research partnerships and that new and traditional data gathering approaches are key to the field of rare plant conservation.

As the editors of the Special Issue, we hope that as you read the papers associated with this Special Issue, you will find that each one of them advances our knowledge and understanding of plant reproductive ecology in various ways, and that the many collaborative efforts occurring for the study of rare plants are improving the field of plant conservation biology.
